# Impaired Lung Function Is Associated with Increased Carotid Intima-Media Thickness in Middle-Aged and Elderly Chinese

**DOI:** 10.1371/journal.pone.0053153

**Published:** 2013-02-15

**Authors:** Zhimin Ma, Yu Liu, Yu Xu, Yun Huang, Min Xu, Xiaolin Zhu, Huijie Zhang, Baihui Xu, Fei Huang, Zhi Yang, Xiaoying Li, Weiqing Wang, Yufang Bi

**Affiliations:** 1 Key Laboratory for Endocrine and Metabolic Diseases of Ministry of Health, Rui-Jin Hospital, Shanghai Jiao Tong University School of Medicine, E-Institute of Shanghai Universities, Shanghai, China; 2 Shanghai Clinical Center for Endocrine and Metabolic Diseases, Shanghai Institute of Endocrine and Metabolic Diseases, Department of Endocrinology and Metabolism, Rui-jin Hospital, Shanghai Jiao Tong University School of Medicine, Shanghai, China; University of Virginia Health System, United States of America

## Abstract

**Background:**

Impairment of lung function was reported to be associated with cardiovascular disease (CVD). The aim of the present study is to evaluate the relationship between lung function and carotid intima-media thickness (cIMT) in participants without chronic pulmonary disease.

**Methodology and Principal Findings:**

A total of 6,423 participants aged 40 years and above were recruited from Jiading District, Shanghai, China. Lung function, evaluated by forced vital capacity (FVC) and forced expiratory volume in one second (FEV1) was measured with standard spirometry. CIMT was measured with high-resolution ultrasonography by trained physicians. Mean values of FVC (% pred) and FEV1 (% pred) in participants with elevated cIMT were significantly lower than in those without (0.92±0.20 vs. 0.99±0.19, 0.83±0.24 vs. 0.90±0.22; both p-values < 0.0001). The levels of cIMT in the lowest quartile of FVC (% pred) and FEV_1_ (% pred) were markedly higher than in the second, third and fourth quartile, respectively (p < 0.0001 for all). The lowest quartile of FVC (% pred) and FEV1 (% pred) was associated with increased odds of elevated cIMT, with the fully adjusted odds ratio of 1.34 and 1.41 (95% confidence interval (CI) 1.09–1.65, p  =  0.006, 95% CI 1.15–1.72, p  =  0.0008), respectively.

**Conclusions and Significance:**

Impaired lung function is associated with elevated cIMT in middle-aged and elderly Chinese. These findings suggest the need to screen impairment of lung function in people without respiratory disease for the presence of subclinical atherosclerosis in CVD prevention.

## Introduction

The traditional risk factors of cardiovascular disease (CVD), such as advancing age, male sex, current smoking, dyslipidemia, diabetes, and hypercholesterolemia, contribute to, but cannot fully explain the increased risk of CVD in the general population. Recently, lung function parameters, estimated by forced vital capacity (FVC) and forced expiratory volume in one second (FEV1) were proved to be well associated with CVD [1]. A number of studies showed that moderate reduction in expiratory flow volumes could increase the risks of ischemic heart disease, heart failure and stroke [2]–[5]. Furthermore, impaired lung function was associated with increased mortality of cardiovascular diseases [6]–[9].

Carotid intima-medial thickness (cIMT) is a sensitive subclinical atherosclerosis marker. An increase of cIMT by 0.1 mm increases the risk of myocardial infarction by 10% to 15% and of stroke by 13% to 18% [10]. Due to the simple and noninvasive assessment of subclinical atherosclerosis, cIMT is well suited for use in large-scale population studies [11]. It was reported that FEV1 was inversely correlated with cIMT in smoking people and community population [12]. However, whether the association can be observed in participants without respiratory disease is still unclear. In addition, it is unclear whether the association can be observed in Chinese population.

The purpose of our study was to investigate the association between lung function and cIMT through estimation of FVC and FEV1 in middle-aged and elderly Chinese.

## Methods

### Ethics statement

This study was approved by the Institutional Review Board of Ruijin Hospital and the written informed consent was obtained from each participant.

### Study population and design

A community-based survey investigating the epidemiology of metabolic diseases and their risk factors was performed in Jiading District, Shanghai, China (March to August, 2010). The study population, design and protocol have been previously described [13]. A total of 10,375 women and men aged 40 years and above were recruited and agreed to participate in this survey. After excluding participants with a history of malignancy, asthma, chronic lung diseases, severe CVD, and incomplete data collection, 6,423 participants with complete data were eventually included in the analysis.

### Clinical and biochemical measurements

The detailed information on demography, medical histories, tobacco use and alcohol consumption, and physical activity was obtained using standard questionnaires by trained physicians. The smoking habit was defined as “non” (never smoking or cessation for equal to and more than 12 months) and “current” (current smoking or cessation for less than 12 months). Current smokers were further grouped into light smokers (0–99 packs a year) and heavy smokers (≥100 packs a year). The alcohol intake habit was recorded as “non” or “current”. Regular exerciser was defined as individual who met the following criteria: 5 or more days of any combination of walking, moderate-intensity or vigorous intensity activities achieving a minimum total physical activity of at least 600 MET-minutes/week [14]. Body weight and height were measured in light clothes and bare feet to the nearest 0.1 kg and 0.1 cm, respectively. Body mass index (BMI) was calculated using the formula of weight/height^2^ (kg/m^2^). Blood pressure was measured on the non-dominant arm in a seated position after a ten-min rest, using an electronic blood pressure monitor (OMRON Model HEM-752 FUZZY, Omron Company, Dalian, China). Three measurements were taken at 1 min interval and the average was used for analysis.

Fasting plasma glucose (FPG), serum total cholesterol (TC), high-density lipoprotein-cholesterol (HDL-c), low-density lipoprotein-cholesterol (LDL-c) and triglycerides (TG) were measured using an automated biochemical instrument (Beckman CX-7 Biochemical Autoanalyser, Brea, CA, USA). Serum insulin levels were measured using radioimmunoassay (Sangon Company, Shanghai, China). The homeostasis model assessment (HOMA) formula, fasting insulin×fasting glucose/22.5, was used to calculate the insulin resistance (IR) score.

### CIMT and lung function measurements

CIMT measurements were performed by a trained sonographer using a high-resolution B-mode tomographic ultrasound system (Esaote Biomedica SpA, Italy) with a linear 7.5 MHz transducer. The measurements were conducted on the far wall of the right and left common carotid arteries, 1.5 cm proximal to the bifurcation. The transducer was manipulated so that the lumen diameter was maximized in the longitudinal plane. CIMT was measured on-line at the end of diastole as the distance from the leading edge of the first echogenic line to that of the second echogenic line. The first and second lines represent the lumen-intimal interface and the collage-contained upper layer of tunic adventitia, respectively. The greater value of the right and left common cIMT was used for analysis.

Lung function tests including FVC and FEV_1_ were conducted by a trained physician using Electronic Spirometer (Model BF-II, Jintan, China) [15]. Each participant received at least two tests (with acceptable maneuvers) at a seated position and with nose clips in place. The predicted values for FVC and FEV_1_ were calculated from the following equations obtained in a representative sample of Chinese population [16].

Predicted FVC of man  = −4.33058–(0.01326× age [years]) + (0.04669× height [cm]) + (0.01664× weight [kg]).

Predicted FVC of woman  = −4.79287– (0.01326× age [years]) + (0.04669× height [cm]) + (0.01664× weight [kg]).

Predicted FEV_1_ of man  = −3.65523– (0.01850× age [years]) + (0.04283× height [cm]) + (0.009228832× weight [kg]).

Predicted FEV_1_ of woman  = −4.04947– (0.01850× age [years]) + (0.04283× height [cm]) + (0.009228832× weight [kg]).

The percentage of predicted values for FEV_1_, FEV_1_ (% pred), equals to FEV_1_ devided by the predicted values of FEV_1_. The percentage of predicted values for FVC, FVC (% pred), equals to FVC devided by the predicted values of FVC. The ratio of FEV_1_ to FVC was calculated.

### Statistical analysis

Statistical analysis was performed using SAS version 9.2 (SAS Institute, Cary, NC). Variables were presented as mean ± standard deviation (SD) for continuous variables and n (%) for categorical variables. TG and HOMA-IR were transformed logarithmically due to non-normal distributions. The cutoff points of FVC (% predicted) quartiles were as follows: quartile 1, <84.7%; quartile 2, 84.7%–97.0%; quartile 3, 97.0%–109.8%; and quartile 4, ≥109.8%. The cutoff points of FEV1 (% predicted) were as follows: quartile 1, <73.9%; quartile 2, 73.9%–89.1%; quartile 3, 89.1%–102.8%; and quartile 4, ≥102.8%. Participants in the highest quintile of cIMT (≥0.7 mm) were classified as having elevated cIMT.

One-way ANOVA for continuous variables and χ^2^-tests for categorical variables were used to access differences among individuals divided according to quartiles of FVC (% predicted), FEV1 (% predicted) and the presence of elevated cIMT, respectively. Simple correlation and multivariate stepwise linear regression analyses were used to evaluate the associations between clinical/biochemical variables and cIMT. Multivariate logistic regression analyses were used to assess the adjusted associations between quartiles of FVC (% predicted), FEV1 (% predicted), and FEV1/FVC ratio and elevated cIMT_._ Age, sex, current smoker, current drinker, regular exerciser, BMI, systolic blood pressure (SBP), diastolic blood pressure (DBP), TC, LDL-c, TG, HDL-c and FPG were included in multivariate logistic regression models as confounders.

The two-tailed test was used and a p value of <0.05 was considered to indicate statistical significance.

## Results

### Clinical and biochemical characteristics

**Figure 1:**
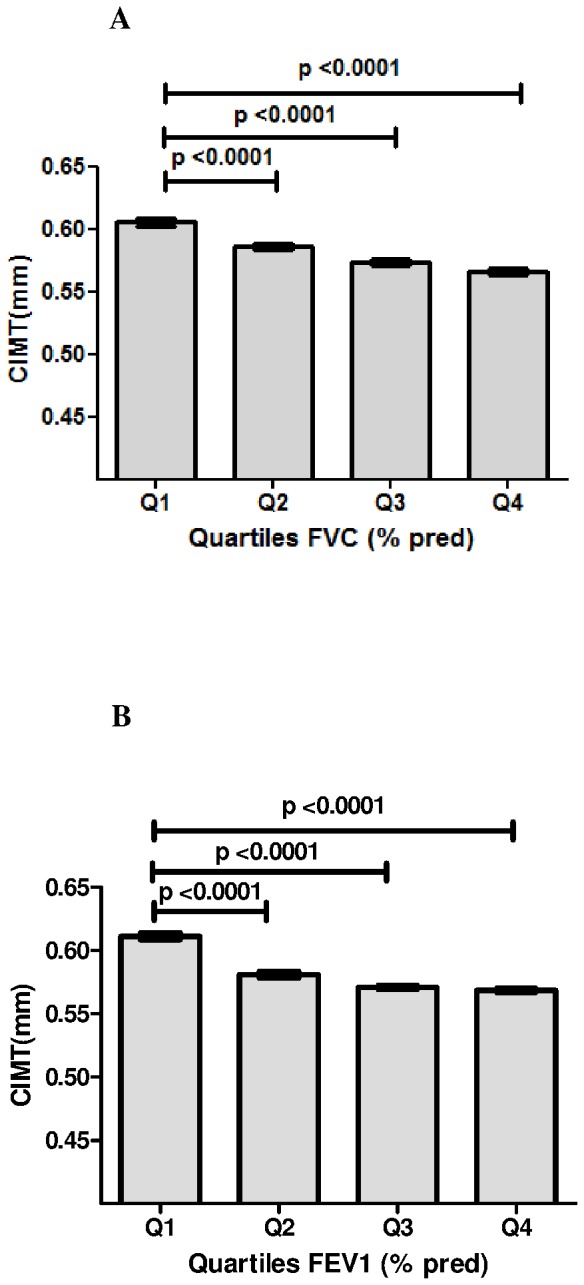
Mean carotid intima-media thickness (cIMT) according to quartiles of FVC (% pred) and FEV_1_ (% pred). A: Mean cIMT according to quartiles (Q) of FVC (% pred). B: Mean cIMT according to quartiles of FEV_1_ (% pred). Data are means ± SD.

As shown in Figure 1, participants in the second, third and highest quartiles of FVC (% pred) and FEV_1_ (% pred) had lower levels of cIMT than those in the lowest ones, respectively (all p values < 0.0001). Mean age, the levels of BMI, SBP, DBP, FPG, HOMA-IR, TG, HDL-c, cIMT and FEV1/FVC ratio, the proportion of male, current drinker, and heavy smoker were statically different across the quartiles of FVC (% predicted) and FEV1 (% predicted), respectively (all p values < 0.05) (Table 1 and in Table 2). The proportion of regular exerciser and the levels of TC, LDL-c were not statistically different across the quartiles of FVC (% predicted) and FEV1 (% predicted), respectively (all p values > 0.05).

**Table 1:** General characteristics according to quartiles of FVC (% predicted).

Variables	Quartile 1 (n = 1584)	Quartile 2 (n = 1619)	Quartile 3 (n = 1607)	Quartile 4 (n = 1613)	p-value
FVC (% predicted)	0.73±0.09	0.91±0.04	1.03±0.04	1.22±0.10	<0.0001
FEV1 (% predicted)	0.68±0.15	0.83±0.15	0.94±0.15	1.10±0.19	<0.0001
FEV1/FVC ratio	0.72±0.13	0.71±0.12	0.71±0.11	0.70±0.10	<0.0001
CIMT (mm)	0.61±0.11	0.58±0.11	0.57±0.10	0.56±0.10	<0.0001
Age (years)	59.6±10.1	57.8±9.3	56.7±8.8	57.4±9.2	<0.0001
Male (n, %)	827 (52.2)	706 (43.6)	531 (33.0)	314 (19.5)	<0.0001
Current drinker (n, %)	202 (12.8)	182 (11.2)	128 (8.0)	74 (4.6)	<0.0001
Smoking status (n, %)					
Non-smoker	1111 (70.1)	1200 (74.1)	1282 (79.8)	1439 (89.2)	<0.0001
Light smoker	38 (2.4)	43 (2.7)	33 (2.1)	8 (0.5)	<0.0001
Heavy smoker	434 (27.4)	375 (23.2)	292 (18.2)	166 (10.3)	<0.0001
Regular exerciser (n, %)	1007 (63.6)	1050 (64.9)	1041 (64.8)	1077 (66.8)	0.30
BMI (kg/m^2^)	26.2±3.4	25.7±3.2	25.1±3.1	24.2±3.0	<0.0001
SBP (mmHg)	145±20	142±20	139±19	138±20	<0.0001
DBP (mmHg)	85±11	84±10	83±10	82±10	<0.0001
FPG (mmol/L)	5.70±1.60	5.60±1.55	5.52±1.42	5.36±1.32	<0.0001
HOMA-IR	1.85 (1.23–2.90)	1.76 (1.21–2.65)	1.67 (1.16–2.47)	1.51 (1.09–2.19)	<0.0001
TG (mmol/L)	1.47 (1.06–2.10)	1.46 (1.06–2.03)	1.38 (0.98–2.02)	1.31 (0.93–1.84)	0.01
TC (mmol/L)	5.37±0.98	5.31±1.02	5.32±0.97	5.35±1.03	0.37
HDL-c (mmol/L)	1.30±0.32	1.28±0.30	1.32±0.32	1.37±0.32	<0.0001
LDL-c (mmol/L)	3.21±0.87	3.18±0.82	3.19±0.85	3.19±0.87	0.5

Data are means ± SD or numbers (percentage) of participants.

P-values for comparisons between groups are based on ANOVA or *χ^2^*-tests.

**Table 2:** General characteristics according to quartiles of FEV1 (% predicted).

Variables	Quartile 1 (n = 1606)	Quartile 2 (n = 1605)	Quartile 3 (n = 1602)	Quartile 4 (n = 1610)	p-value
FEV1 (% predicted)	0.61±0.10	0.82±0.04	0.96±0.04	1.17±0.13	<0.0001
FVC (% predicted)	0.80±0.16	0.92±0.13	1.01±0.11	1.17±0.14	<0.0001
FEV1/FVC ratio	0.60±0.12	0.71±0.10	0.75±0.08	0.77±0.08	<0.0001
CIMT (mm)	0.61±0.11	0.58±0.11	0.57±0.10	0.57±0.09	<0.0001
Age (years)	59.7±9.8	57.0±9.3	56.6±9.0	58.1±9.3	<0.0001
Male (n, %)	757 (47.1)	680 (42.4)	600 (37.5)	341 (21.2)	<0.0001
Current drinker (n, %)	189 (11.8)	164 (10.2)	150 (9.4)	83 (5.2)	<0.0001
Smoking status (n, %)					
Non-smoker	1173 (73.0)	1216 (75.8)	1227 (76.6)	1416 (88.0)	<0.0001
Light smoker	43 (2.7)	35 (2.2)	30 (1.9)	11 (0.9)	<0.0001
Heavy smoker	388 (24.2)	354 (22.1)	345 (21.5)	180 (11.2)	<0.0001
Regular exerciser (n, %)	1050 (65.4)	1029 (64.1)	1039 (64.9)	1057 (65.7)	0.81
BMI (kg/m^2^)	25.7±3.3	25.5±3.4	25.2±3.1	24.8±3.0	<0.0001
SBP (mmHg)	143±20	140±20	140±20	140±20	<0.0001
DBP (mmHg)	84±10	84±10	83±10	82±10	<0.0001
FPG (mmol/L)	5.61±1.50	5.60±1.60	5.52±1.47	5.44±1.35	0.004
HOMA-IR	1.73 (1.19–2.69)	1.73 (1.19–2.63)	1.66 (1.13–2.51)	1.60 (1.15–2.37)	<0.0001
TG (mmol/L)	1.44 (1.03–2.02)	1.41 (1.02–2.01)	1.41 (1.00–2.00)	1.37 (0.97–1.93)	0.03
TC (mmol/L)	5.36±0.95	5.31±1.03	5.32±1.00	5.36±1.01	0.38
HDL-c (mmol/L)	1.31±0.31	1.30±0.32	1.30±0.31	1.35±0.32	<0.0001
LDL-c (mmol/L)	3.22±0.85	3.17±0.83	3.18±0.86	3.21±0.87	0.26

Data are means ± SD or numbers (percentage) of participants.

P-values for comparisons between groups are based on ANOVA or *χ^2^*-tests.

Mean values of FVC (% pred), FEV1 (% pred) and FEV1/FVC ratio in participants with elevated cIMT were significantly lower than in those without (0.92±0.20 vs. 0.99±0.19, 0.83±0.24 vs. 0.90±0.22, 0.68±0.12 vs. 0.72±0.11; both p < 0.0001). Individuals with elevated cIMT were elder, more likely to be men, current drinker, and heavy smoker, and had higher levels of BMI, SBP, DBP, FPG, HOMA-IR, TG, TC, LDL-c, and HDL-c (all p values < 0.05) (Table 3).

**Table 3:** General characteristics according to the presence of elevated cIMT.

	Non-elevated cIMT (n = 5140)	Elevated cIMT (n = 1283)	p-value
CIMT (mm)	0.54±0.07	0.74±0.08	<0.0001
FVC (% pred)	0.99±0.19	0.92±0.20	<0.0001
FEV_1_ (% pred)	0.90±0.22	0.83±0.24	<0.0001
FEV1/FVC ratio	0.72±0.11	0.68±0.12	<0.0001
Age (years)	56.2±8.7	64.3±9.4	<0.0001
Male (n, %)	1658 (32.3)	720(56.1)	<0.0001
Smoking status (n, %)			
Non-smoker	4138 (80.5)	894 (69.7)	<0.0001
Light smoker	83 (1.6)	39 (3.0)	<0.0001
Heavy smoker	919 (17.9)	348 (27.1)	<0.0001
Current drinker (n, %)	419 (8.2)	167 (13.0)	<0.0001
Regular exerciser (n, %)	3280 (63.8)	895 (69.8)	<0.0001
BMI (kg/m^2^)	25.1±3.2	26.1±3.3	<0.0001
SBP (mmHg)	138±19	149±20	<0.0001
DBP (mmHg)	83±10	84±11	0.002
FPG (mmol/L)	5.46±1.38	5.86±1.80	<0.0001
HOMA-IR	1.64 (1.12–2.44)	1.86 (1.15–2.81)	<0.0001
TG (mmol/L)	1.38 (0.98–1.97)	1.47 (1.05–2.05)	0.005
TC (mmol/L)	5.30±1.00	5.48±1.00	<0.0001
LDL-c (mmol/L)	3.15±0.84	3.35±0.87	<0.0001
HDL-c (mmol/L)	1.32±0.31	1.29±0.32	<0.0001

Data are means ± SD or numbers (percentage) of participants.

P-values for comparisons between groups are based on ANOVA or *χ^2^*-tests.

### Risk factors related to cIMT

Simple correlation analyses revealed that age, gender, smoking status, BMI, SBP, DBP, TG, TC, LDL-c, HDL-c, HOMA-IR, FVC (% pred), FEV1 (% pred), and FEV1/FVC ratio were associated with cIMT. In multiple stepwise linear regression models, FVC (% pred), FEV_1_ (% pred) and FEV1/FVC ratio were independently associated with cIMT, besides age, gender, smoking status, BMI, SBP, DBP, LDL-c, HDL-c (Table 4).

**Table 4:** Simple correlation and multiple stepwise linear regression analysis of risk factors associated with cIMT.

	Simple		Stepwise†		Stepwise‡		Stepwise§	
	*r*	p-value	*β*	p-value	*β*	p-value	*β*	p-value
FVC (% pred)	−0.15	<0.0001	−0.03	0.02	−	−	−	−
FEV_1_ (% pred)	−0.14	<0.0001	−	−	−0.07	<0.0001	−	−
FEV1/FVC ratio	−0.15	<0.0001	−	−	−	−	−0.08	<0.0001
Age (years)	0.45	<0.0001	0.36	<0.0001	0.36	<0.0001	0.35	<0.0001
Gender (male = 1, female = 0)	−0.24	<0.0001	−0.20	<0.0001	−0.19	<0.0001	−0.21	<0.0001
Current smoker (yes = 1, no = 0)	0.13	<0.0001	0.05	<0.0001	0.05	<0.0001	0.06	<0.0001
BMI (kg/m^2^)	0.13	<0.0001	0.07	<0.0001	0.07	<0.0001	0.08	<0.0001
SBP (mmHg)	0.26	<0.0001	0.14	<0.0001	0.14	<0.0001	0.15	<0.0001
DBP (mmHg)	0.03	0.005	−0.09	<0.0001	−00.1	<0.0001	−0.09	<0.0001
TG (mmol/L)*	0.054	<0.0001	−0.03	0.156	−0.03	0.17	−0.02	0.57
TC (mmol/L)	0.10	<0.0001	0.001	0.980	0.000	1.00	0.002	0.94
LDL-c (mmol/L)	0.09	<0.0001	0.12	<.0001	0.12	<0.0001	0.114	<0.0001
HDL-c (mmol/L)	−0.053	<0.0001	−0.03	0.05	−0.03	0.05	−0.03	0.05
HOMA-IR*	0.07	<0.0001	0.02	0.08	0.02	0.10	0.003	0.05

r, correlation coefficient; β, regression coefficient; SE, standard error. *: log-transfer. †: FVC (% pred) involved. ‡: FEV1 (% pred).

§ : FEV1/FVC ratio involved.

### Association between quartiles of FVC (% pred) and FEV1 (% pred) and cIMT

As shown in Table 5, the lowest quartile of FVC (% pred) and FEV1 (% pred) was associated with increased odds of elevated cIMT, with age- and sex-adjusted odds ratio (OR) of 1.62 and 1.53, respectively (95% confidential interval (CI), 1.33–1.97 and 95% CI, 1.26–1.85; both p < 0.0001). Further adjustments for current smoker, current drinker, regular exerciser, TC, LDL-c, TG, HDL-c, FPG, SBP, DBP and BMI did not eliminate the associations (OR, 1.34, 95% CI, 1.09–1.65, p  =  0.006 and OR,1.41, 95% CI, 1.15–1.72, p  =  0.0008). The lowest quartile of FEV1/FVC ratio was also associated with increased odds of elevated cIMT (fully-adjusted OR, 1.46, 95% CI, 1.20–1.78, p<0.0001).

**Table 5:** Odds ratio for the presence of elevated cIMT according to quartiles of FVC (% pred) or FEV_1_ (% pred).

	Model 1		Model 2		Model 3	
	OR (95% CI)	p-value	OR (95% CI)	p-value	OR (95% CI)	p-value
FVC (% pred)						
Quartile 1	1.62 (1.33–1.97)	<0.0001	1.49 (1.21–1.83)	0.0001	1.34 (1.09–1.65)	0.006
Quartile 2	1.25 (1.02–1.54)	0.03	1.15 (0.93–1.42)	0.19	1.07 (0.87–1.33)	0.52
Quartile 3	1.12 (0.91–1.38)	0.29	1.04 (0.84–1.30)	0.70	1.01 (0.81–1.25)	0.96
Quartile 4	1.00	–	1.00	–	1.00	–
FEV_1_ (% pred)						
Quartile 1	1.53 (1.26–1.85)	<0.0001	1.49 (1.22–1.82)	<0.0001	1.41 (1.15–1.72)	0.0008
Quartile 2	1.28 (1.05–1.56)	0.02	1.24 (1.01–1.52)	0.04	1.18 (0.96–1.45)	0.12
Quartile 3	0.91 (0.74–1.12)	0.35	0.86 (0.69–1.07)	0.17	0.84 (0.68–1.04)	0.11
Quartile 4	1.00	–	1.00	–	1.00	–
FEV1/FVC ratio						
Quartile 1	1.44 (1.19–1.75)	<0.0001	1.47 (1.20–1.79)	<0.0001	1.46 (1.20–1.78)	<0.0001
Quartile 2	1.14 (0.93–1.39)	0.20	1.13 (0.92–1.39)	0.25	1.12 (0.91–1.37)	0.30
Quartile 3	0.86 (0.70–1.06)	0.16	0.84 (0.68–1.04)	0.11	0.82 (0.67–1.02)	0.08
Quartile 4	1.00	–	1.00	–	1.00	–

The cutoff points of FEV1/FVC ratio were as follows: quartile 1, <64.0%; quartile 2, 64.0%–72.0%; quartile 3, 72.0%–79.0%; and quartile 4, ≥79.0%.

OR, odd ratio; 95% CI, 95% confidence interval.

Model 1:Adjusted for age, sex;

Model 2:Model 1 covariates plus current smoker, current drinker, regular exerciser, TC, LDL-c, TG, HDL-c, FPG, SBP and DBP;

Model 3:Model 2 covariates plus BMI.

In never smokers, the lowest quartile of FVC (% pred) and FEV1 (% pred) was associated with increased risk of elevated cIMT, respectively (fully-adjusted OR, 1.28, 95% CI, 1.01–1.63, p  =  0.006 and fully-adjusted OR, 1.51, 95% CI, 1.19–1.90, p < 0.0001) (Table S1). In current smokers, the lowest quartile of FEV1 (% pred) was associated with increased risk of elevated cIMT (fully-adjusted OR, 1.66, 95% CI, 1.15–2.42, p  =  0.007) (Table S2), whereas the similar trend was not found between quartiles of FEV1 (% pred) and cIMT.

## Discussion

In the present study, we found that impaired lung function, estimated by FVC (% pred) and FEV1 (% pred) was associated with elevated cIMT in a middle-aged population without chronic pulmonary diseases. Multiple logistic regression analyses showed that the OR of elevated cIMT in participants in the lowest quartiles of FVC (% pred) and FEV_1_ (% pred) was increased by 34% and 41%, respectively. Adjustments for age, sex and other potential traditional risk factors did not eliminate the association, indicating an independent relationship between impaired lung function and elevated cIMT in a middle-aged Chinese. In non-smokers, the associations were still statically significant in full adjustment models.

The association between lung function and subclinical atherosclerosis is still controversial, probably due to the definition of impairment of lung function and subclinical atherosclerosis and the study design [12], [17]. We found in a community-based population that independent of potential confounding factors, the lowest quartiles of FEV1 (% pred) and FEV_1_ (% pred) were associated with elevated cIMT. In addition, individuals with asthma, chronic lung diseases were not involved in the final analysis, the need to screen impairment of lung function in participants without respiratory disease for the presence of subclinical atherosclerosis in CVD prevention.

In the cross-sectional analysis of the ARIC study, decreased FEV1 was associated with increased cIMT in current smokers; however, further adjustment for CVD risk factors eliminated the association in never smokers [12]. Nevertheless, in a retrospective study of Korean males, smoking status did not affect the significant associations between quartiles of FVC (% pred) and FEV1 (% pred) and cIMT; although traditional CVD risk factors not involved in adjustment models, except age, smoking status and BMI [17]. In the present study, even after adjustment for a variety of potentially confounders, the associations between FVC (% pred), FEV1 (% pred) and cIMT in the whole population and never smokers were still statically significant. In current smokers, decreased FEV1 (% pred) was associated with increased cIMT. Decreased FVC (% pred) was associated with increased cIMT, adjusted for age, sex, but further adjustment for SBP, FPG, and BMI eliminated the association. It was presumed that these factors were involved in the association in current smokers. These findings suggested a complex relationship between lung function and atherosclerotic vascular disease that invites further study.

Several possible mechanisms account for our findings of the association between lung function and subclinical atherosclerosis, including HOMA-IR, obesity and other “established” cardiovascular risk factors [18]–[20]. Consistent with the previous study [6], our results showed that insulin resistance and BMI were significantly increased both in participants with poor lung function and with increased cIMT, demonstrating the role of insulin resistance and BMI in the association. Adjustment for BMI in the multiple logistic regression models attenuated, but did not eliminate the association. These findings indicated that other factors might be involved in the association between lung function and subclinical atherosclerosis. Systematic inflammation induced by impaired lung function could be a causal factor for atherosclerosis [21]–[23]. In addition, the imbalance between the demand and the supply of oxygen in the arterial wall for reduced lung function was suggested to be a key factor in the development of atherosclerotic lesions [24], [25]. However, the measurement of markers of inflammation and chronic hypoxemia was absent, limiting the ability to access the role of these factors in the association in the present study.

The limitations of our present study should be taken into account. First, this was a cross-sectional study; therefore, a causal link between impaired lung function and cIMT could not be drawn. Secondly, smoking status might be a confounder of the association. However, the limited sample size of light smokers in each quartile group of FVC limited the ability to access the interaction of current smoking categories with lung function on the percentage of elevated cIMT. Thirdly, residual confounding by unmeasured variables cannot be excluded, despite detailed adjustment for risk factors for subclinical atherosclerosis, such as passive smoking.

In conclusion, impaired lung function is associated with elevated cIMT in middle-aged and elderly Chinese population. These findings suggest the need to screen impairment of lung function in people without respiratory disease for the presence of subclinical atherosclerosis in CVD prevention.

## Supporting Information

Table S1 Odds ratio for the presence of elevated cIMT according to quartiles of FVC (% pred) or FEV_1_ (% pred) in non-smokers.
(DOC)

Table S2 Odds ratio for the presence of elevated cIMT according to quartiles of FVC (% pred) or FEV_1_ (% pred) in smokers (n = 1391).
(DOC)
