# Supplementary material for: Impaired Lung Function Is Associated with Increased Carotid Intima-Media Thickness in Middle-Aged and Elderly Chinese
Source: PLoS One. 2013 Feb 15;8(2):e53153. doi: 10.1371/journal.pone.0053153 (PMC3574141; doi:10.1371/journal.pone.0053153)
Supplement: Table S1 — Odds ratio for the presence of elevated cIMT according to quartiles of FVC (% pred) or FEV1 (% pred) in non-smokers. (DOC) [file pone.0053153.s001.doc]

**Table S1. Odds ratio for the presence of elevated cIMT according to quartiles of FVC (% pred) or FEV_1_ (% pred) in non-smokers**

|  | Model 1 |  |  | Model 2 |  |  | Model 3 |  |
| --- | --- | --- | --- | --- | --- | --- | --- | --- |
|  | OR (95% CI) | P Value |  | OR (95% CI) | P Value |  | OR (95% CI) | P Value |
| FVC (% pred) |  |  |  |  |  |  |  |  |
| Quartile 1 | 1.55 (1.23–1.94) | <0.0001 |  | 1.41 (1.12–1.79) | 0.004 |  | 1.28 (1.01–1.63) | 0.006 |
| Quartile 2 | 1.21 (0.96–1.53) | 0.11 |  | 1.13 (0.89–1.44) | 0.33 |  | 1.05 (0.82–1.35) | 0.67 |
| Quartile 3 | 1.01 (0.79–1.29) | 0.93 |  | 0.94 (0.73–1.21) | 0.61 |  | 0.90 (0.70–1.17) | 0.43 |
| Quartile 4 | 1.00 | – |  | 1.00 | – |  | 1.00 | – |
| FEV1 (% pred) |  |  |  |  |  |  |  |  |
| Quartile 1 | 1.68 (1.34–2.10) | <0.0001 |  | 1.59 (1.26–2.00) | <0.0001 |  | 1.51 (1.19–1.90) | <0.0001 |
| Quartile 2 | 1.37 (1.09–1.74) | 0.008 |  | 1.32 (1.04–1.68) | 0.03 |  | 1.27 (0.99–1.62) | 0.06 |
| Quartile 3 | 1.07 (0.84–1.37) | 0.56 |  | 0.98 (0.77–1.27) | 0.90 |  | 0.96 (0.75–1.24) | 0.78 |
| Quartile 4 | 1.00 | – |  | 1.00 | – |  | 1.00 | – |

OR, odd ratio; 95% CI, 95% confidence interval.

Model 1：Adjusted for age, sex;

Model 2：Model 1 covariates plus current drinker, regular exerciser, TC, LDL-c, TG, HDL-c, FPG, SBP and DBP;

Model 3：Model 2 covariates plus BMI
