# Supplementary material for: Impaired Lung Function Is Associated with Increased Carotid Intima-Media Thickness in Middle-Aged and Elderly Chinese
Source: PLoS One. 2013 Feb 15;8(2):e53153. doi: 10.1371/journal.pone.0053153 (PMC3574141; doi:10.1371/journal.pone.0053153)
Supplement: Table S2 — Odds ratio for the presence of elevated cIMT according to quartiles of FVC (% pred) or FEV1 (% pred) in smokers (n = 1391). (DOC) [file pone.0053153.s002.doc]

**Table S2.** Odds ratio for the presence of elevated cIMT according to quartiles of FVC (% pred) or FEV_1_ (% pred) in smokers (n=1391)

|  | Model 1 | |  | Model 2 | |  | Model 3 | |
| --- | --- | --- | --- | --- | --- | --- | --- | --- |
|  | OR (95% CI) | *P* Value |  | OR (95% CI) | *P* Value |  | OR (95% CI) | *P* Value |
| FVC (% pred) | |  |  |  |  |  |  |  |
| Quartile 1 | 1.52 (1.07–2.19) | 0.02 |  | 1.41 (0.97–2.06) | 0.07 |  | 1.26 (0.86–1.85) | 0.24 |
| Quartile 2 | 1.31 (0.91–1.89) | 0.14 |  | 1.22 (0.84–1.79) | 0.33 |  | 1.15 (0.79–1.69) | 0.46 |
| Quartile 3 | 1.02 (0.70–1.48) | 0.93 |  | 0.91 (0.62–1.34) | 0.63 |  | 0.87 (0.59–1.29) | 0.49 |
| Quartile 4 | 1.00 | – |  | 1.00 | – |  | 1.00 | – |
| FEV_1_ (% pred) | |  |  |  |  |  |  |  |
| Quartile 1 | 1.74 (1.22–2.48) | 0.002 |  | 1.75 (1.21–2.52) | 0.003 |  | 1.66 (1.15–2.42) | 0.007 |
| Quartile 2 | 1.28 (0.89–1.85) | 0.18 |  | 1.21 (0.82–1.77) | 0.34 |  | 1.23 (0.77–1.66) | 0.54 |
| Quartile 3 | 0.97 (0.66–1.42) | 0.88 |  | 0.96 (0.65–1.43) | 0.85 |  | 0.94 (0.63–1.39) | 0.74 |
| Quartile 4 | 1.00 | – |  | 1.00 | – |  | 1.00 | – |

OR, odd ratio; 95% CI, 95% confidence interval.

Model 1：Adjusted for age, sex;

Model 2：Model 1 covariates plus current drinker, regular exerciser, TC, LDL-c, TG, HDL-c, FPG, SBP and DBP;

Model 3：Model 2 covariates plus BMI.
